# Classification and treatment of lateral malleolar fractures - a single-center analysis of 439 ankle fractures using the Swedish Fracture Register

**DOI:** 10.1186/s12891-020-03542-5

**Published:** 2020-08-05

**Authors:** Emilia Möller Rydberg, Tina Zorko, Mikael Sundfeldt, Michael Möller, David Wennergren

**Affiliations:** 1 Institute of Clinical Sciences, Sahlgrenska Academy, University of Gothenburg, Gothenburg, Sweden; 2 Department of Orthopaedics, Sahlgrenska University Hospital, Göteborgsvägen 31, SE-431 80 Gothenburg/Mölndal, Sweden

**Keywords:** Ankle fracture, Swedish fracture Register, Epidemiology, Fracture management

## Abstract

**Background:**

The decision regarding which trans syndesmotic ankle fractures to treat surgically and which to treat non-surgically is a matter of debate. The aim of this study was to describe the epidemiology of ankle fractures treated at Sahlgrenska University Hospital (SU) during a 2-year period and analyze the current diagnostic process, classification and choice of treatment for lateral malleolar fractures at the level of the syndesmosis.

**Methods:**

Observational data regarding all ankle fractures treated at SU between 1 April 2012 and 31 March 2014 was collected from the Swedish Fracture Register. For identified AO/OTA44-B1 fractures, medical records and radiographs were reviewed and analyzed.

**Results:**

The study included 1332 ankle fractures. 838 (63%) were B-fractures and 512 (38%) of these were B1 fractures. 439 of the patients with B1 fractures were included in the detailed study and of these 309 (70%) were treated non-surgically and 130 patients (30%) surgically. According to the preoperative physical examination described in the medical records, medial tenderness was found in 73 (24%) of the non-surgically treated patients. Among the surgically treated patients 18% (*n* = 24) were found to have no medial tenderness. For the non-surgically treated patients with medial tenderness, the treatment plan was changed to surgical treatment after the 1-week radiographic follow-up in 1 patient (1%) and 1 patient (1%) was treated surgically after 3 months due to non-union.

**Conclusions:**

The current study demonstrates the difficulty involved in distinguishing whether or not a trans-syndesmotic lateral malleolar fracture has an associated medial ligament injury or not. As this distinguishes if the fracture is stable or not it affects the choice of subsequent treatment. The results of the study also indicate a lack of consensus on how to classify and treat lateral malleolar fractures at the level of the syndesmosis. The study further suggests that there is no need to check non-surgically treated stable fractures with follow-up radiographic examination at 1 week.

## Background

The Swedish Fracture Register (SFR) is a national quality register that collects data on all types of fracture regarding cause of injury, type of fracture, given treatment, and subsequent treatment results [1]. Several studies have been conducted in order to validate the data in the register and these studies have shown that the SFR has acceptable validity [2–5]. The SFR provides a unique opportunity to study the epidemiology of fractures [6]. Thur et al. studied the epidemiology of ankle fractures in Sweden between 1987 and 2004 but there is a lack of up to date epidemiological studies on ankle fractures [7].

**Fig. 1:**
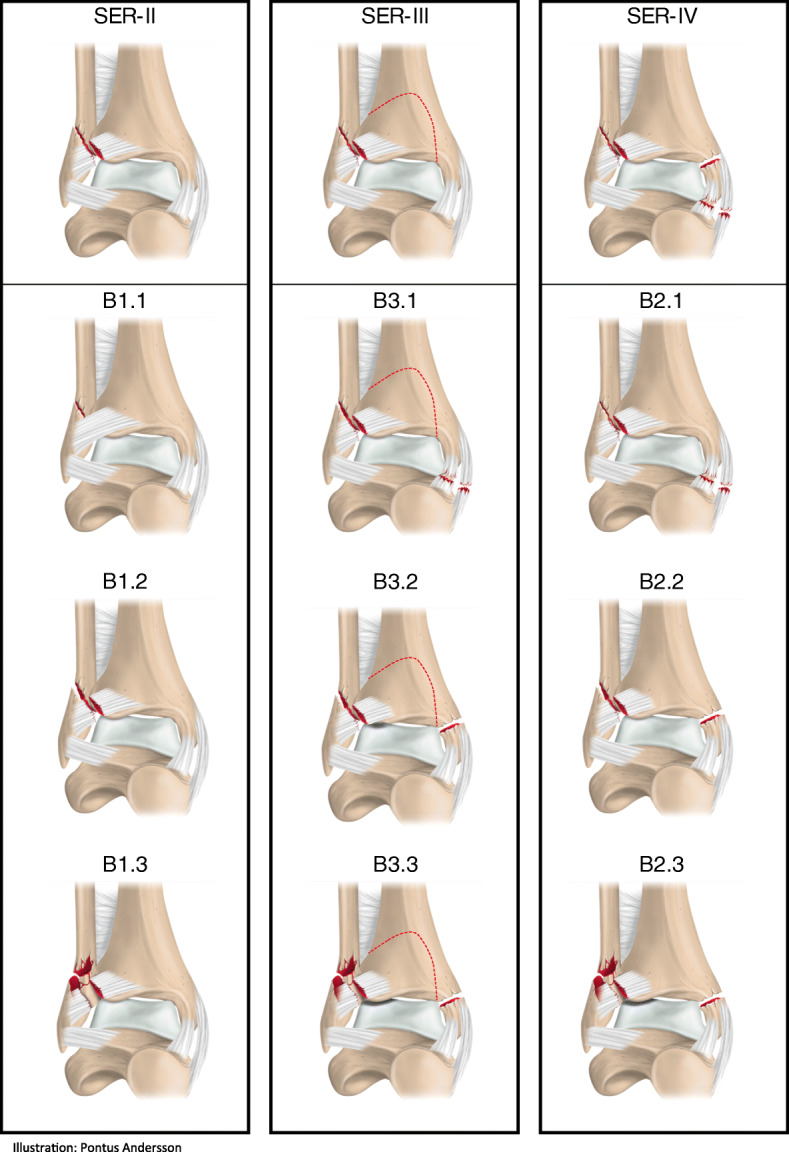
The AO/OTA-classification of ankle fractures compared with the Lauge-Hansen classification of SER-injuries (supination-external rotation)

Lateral malleolar fractures at the level of the syndesmosis, AO/OTA 44 B fractures, are the fourth most common fracture registered in the SFR and are commonly referred to as the equivalent of Lauge-Hansen supination-external rotation (SER) injuries [8, 9] (Fig. 1). Both a lateral malleolar fracture without an impaired deltoid ligament (SER-II or B1) and the combination of a lateral malleolar fracture with a deltoid ligament rupture (SER-IV or B2) appear as unimalleolar on plain radiographs (Fig. 1).

The isolated lateral fracture (B1/SER-II) is by nature stable, suitable for non-surgical treatment and should not be exposed to the risk of complications due to surgery [10–12]. On the other hand, the B2 or SER-IV fracture with a fracture pattern that proceeds to the medial side is possibly unstable and better suited to surgical treatment [13, 14]. Since the two fracture types might appear similar on plain radiographs, the physical examination of the patient is a crucial part in the decision-making regarding treatment method. The best way to determine whether or not a lateral malleolar fracture at the level of the syndesmosis has an associated deltoid ligament injury has not been fully elucidated. Despite the fact that it’s been shown that medial tenderness, brusing or ecchymosis are unreliable predictors of medial ligament injury these are the signs used in clinical practice today.

The aim of the current study was to give an up-to-date description of the epidemiology of ankle fractures. The aim was also to conduct a deeper analysis of the current clinical diagnostic process regarding injury to the deltoid ligament, subsequent classification and the choice of treatment method for lateral malleolar fractures at the level of the syndesmosis.

## Methods

**Fig. 2:**
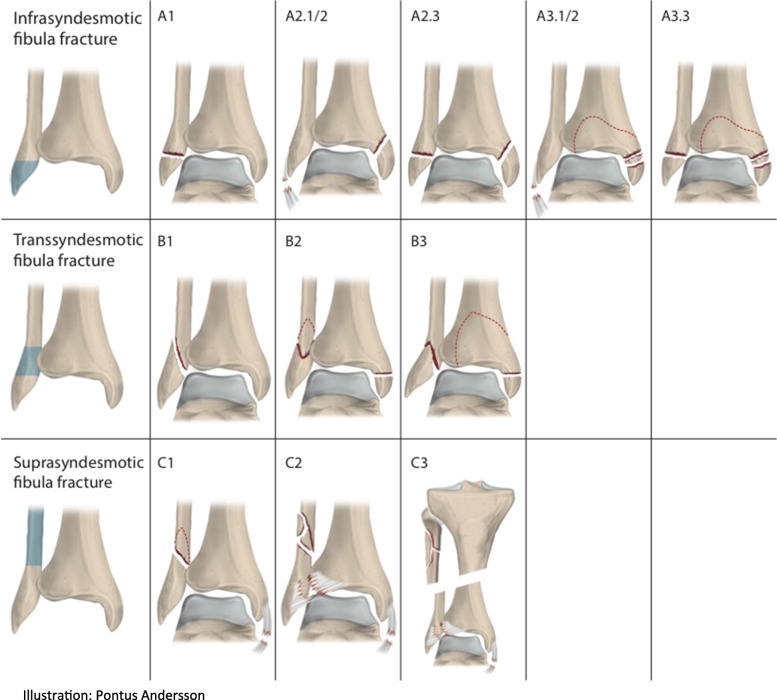
The AO/OTA-classification of ankle fractures

**Fig. 3:**
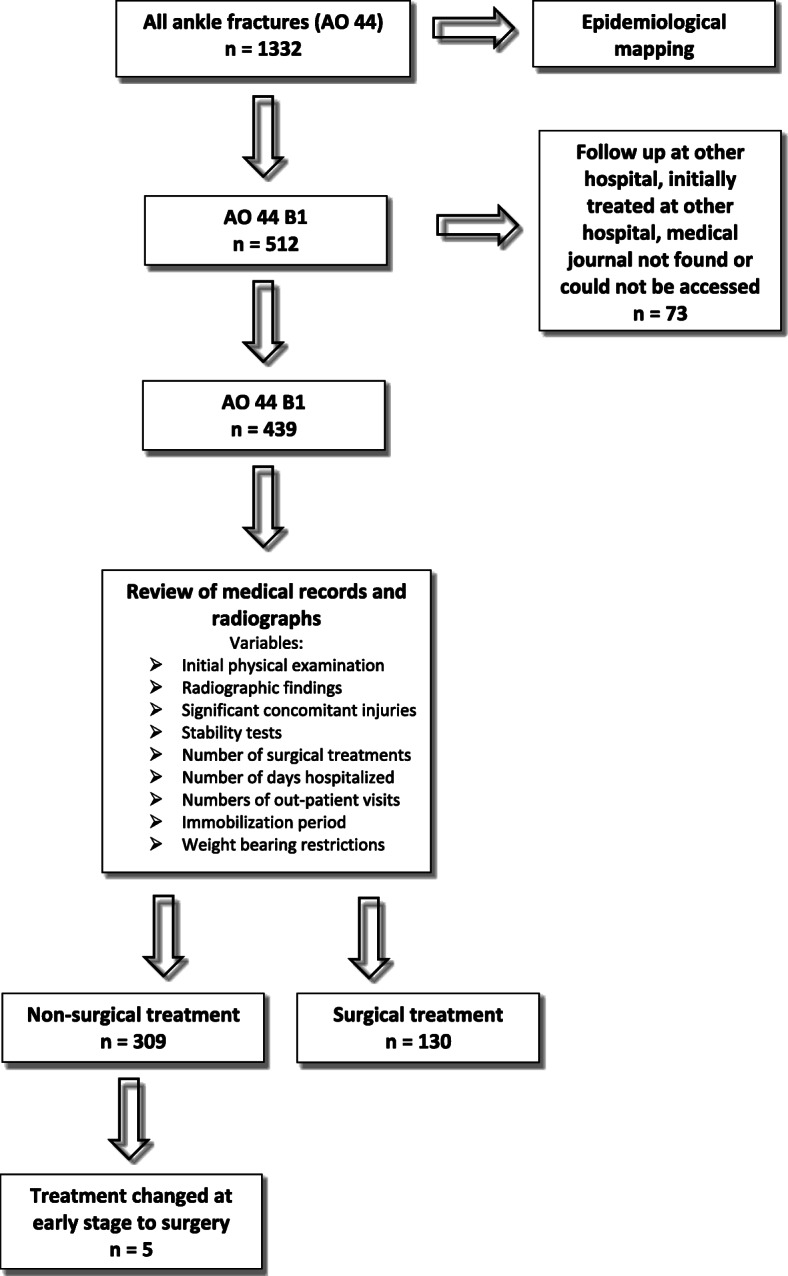
Flow chart of how the study was conducted

For all patients registered at Sahlgrenska University Hospital (SU) with an ankle fracture between 1 April 2012 and 31 March 2014, data were collected from the SFR. In SFR ankle fractures are classified according to the Arbeitsgemeinschaft für Osteosynthesefragen (AO)/Orthopaedic Trauma Association (OTA) classification [15, 16] (Fig. 2). An analysis of data from the SFR relating to fracture classification, age, sex, cause of injury, frequency of high−/low-energy trauma and frequency of closed/open fracture was conducted. In addition to the observational data from the SFR, medical records and radiographs were reviewed for all patients registered with an AO/OTA 44 B1 fracture (Fig. 3). From the medical records information regarding signs of medial ligament injuries found at the initial physical examination was obtained as well as instructions regarding weight bearing restrictions and immobilization period. The number of outpatient visits, days hospitalized and number of radiographic examinations performed were counted. From medical records it was noted if stability tests had been performed and if there were any significant concomitant injuries. Radiographs were reviewed in cases were treatment deviated from standard care to make sure the fracture classification was correct. The medical records of the patients that had initially been assigned to non-surgical treatment but had been changed to surgical treatment at an early stage were additionally studied to ascertain why the treatment strategy had been changed.

### Statistical methods

Descriptive statistical analyses and the building of charts were executed in Excel 2013.

## Results

During the 2 consecutive years from 1 April 2012 and 31 March 2014, 1328 patients with ankle fractures were treated at SU. Four of the patients had sustained 2 ankle fractures and, as a result, the total number of ankle fractures treated at SU during this period was 1332. Of these, 512 were classified as B1 fractures, of which 439 were both initially treated and followed up at SU and were included in the detailed study. For the remaining 73 patients initial treatment or follow up was conducted at another hospital or medical records was not found or could not be accessed therefore they were not included in the study (Fig. 3).

### Epidemiology of ankle fractures

As shown in Table 1, 27% of the ankle fractures were infra-syndesmotic type A fractures, 63% were trans-syndesmotic type B fractures and 11% were supra-syndesmotic type C fractures.

**Table 1:** Demographics of patients registered with ankle fracture at Sahlgrenska University Hospital between 2012-04-01 and 2014-03-31. Some cases had missing information regarding high or low energy, therefore the sum is not 100%

		A *N* = 354 (27%)			B *N* = 838 (63%)			C *N* = 140 (11%)		
		A1 *N* = 225 (64%)	A2 *N* = 109 (31%)	A3 *N* = 20 (6%)	B1 *N* = 512 (61%)	B2 *N* = 158 (19%)	B3 *N* = 168 (20%)	C1 *N* = 58 (41%)	C2 *N* = 36 (26%)	C3 *N* = 46 (33%)
Sex	Male, N (%)	80 (36)	47 (43)	13 (65)	245 (48)	56 (35)	51 (30)	24 (41)	9 (25)	35 (76)
	Female, N (%)	145 (64)	62 (57)	7 (35)	267 (52)	102 (65)	117 (70)	34 (59)	27 (75)	11 (24)
Mean age	Total, years (range)	50.4 (16–93)	51.8 (16–96)	48 (20–95)	51.8 (16–98)	58.4 (18–98)	58.4 (17–94)	47.9 (16–97)	51.9 (18–90)	46.8 (17–86)
	Male, years (range)	41.8 (16–88)	47.4 (16–96)	42.6 (21–95)	47.7 (16–90)	53.6 (18–95)	52.7 (17–86)	40.8 (17–82)	37.9 (21–61)	45.7 (17–86)
	Female, years (range)	55.2 (16–93)	55.2 (16–91)	57.9 (20–95)	55.6 (16–98)	60.9 (19–98)	60.9 (22–94)	52.9 (16–97)	56.6 (18–90)	50.5 (24–81)
Fracture	Open, N (%)	1 (0.4)	4 (3.7)	0 (0)	6 (1.2)	2 (1.3)	4 (2.4)	0 (0)	3 (8.3)	2 (4.3)
	High energy, N (%)	7 (3.1)	21 (19.2)	1 (5)	17 (3.3)	8 (5.1)	8 (4.8)	4 (6.9)	5 (13.9)	5 (10.9)
	Low energy, N (%)	213 (94.7)	84 (77.1)	18 (90)	488 (95.3)	150 (95)	157 (93.5)	52 (89.7)	31 (86.1)	41 (89.1)

For the group comprising all ankle fractures, there was a slight predominance of women (men: women 42:58). This pattern was also seen for most of the individual fracture classes with the exception of the groups of A3 and C3 fractures where there were substantially more men than women (A3 men: women 65:35 and C3 men: women 76:24) (Table 1).

In the study population comprising all ankle fractures, the median age was 55 years (16–98) and the men had a mean age of 47 years and the women 57 years. The most commonly affected age group was 56–65 years. Of the patients with C2 fractures, 75% were women and their mean age was 57 years, whereas the men in the same group had a mean age of 38 years (Table 1). Among the patients with C3 fractures, the mean age was 47 years and 76% of the patients were men (Table 1).

Of all ankle fractures, 93% were due to low-energy trauma and 98% were closed fractures. In the A2 group, 19% of the fractures were due to high-energy trauma and 8% of the C2 group were open fractures (Table 1).

The most common cause of injury was a fall on the same level due to slipping, tipping or stumbling (47%) for all ankle fractures, as well as for all subtypes respectively. 26% of the A2 fractures were due to vehicle accidents and 20% of the C3 fractures were due to slipping on ice and/or snow.

### AO/OTA 44 B1 fractures

Among the patients registered in the SFR as having sustained a B1 fracture, the sex distribution was 47% men and 53% women. The total mean age was 53 years, with a slightly higher mean age among the women (men: women 49:56). 96% of the fractures were due to low-energy trauma and 99% were closed injuries. 47% of the fractures were caused by a fall on the same level due to slipping, tripping or stumbling.

**Fig. 4:**
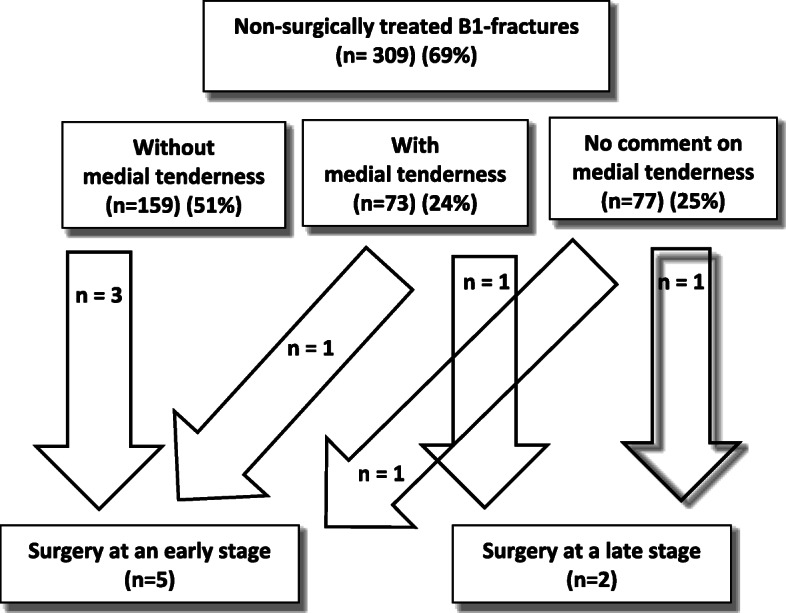
Flow chart of how the AO/OTA 44B1 fractures that were initially assigned to non-surgical treatment were followed up and eventually treated

Among the 439 patients registered with B1 fractures, 309 patients (70%) were treated non-surgically and 130 (30%) surgically. According to the preoperative physical examination described in the medical records, medial tenderness was found in 73 (24%) of the non-surgically treated patients and in 77 (25%) there was no comment on medial tenderness in the medical charts. Among the surgically treated patients with B1 fractures, 62 (48%) of the patients had medial tenderness and in 44 (34%) patients no comment was made on medial tenderness. 18% (*n* = 24) of the surgically treated patients were found to have no medial tenderness. At our department all non-surgically treated B1 fractures are followed up at 1 week with a radiographic examination. For the 73 (24%) non-surgically treated patients with medial tenderness, the treatment plan was changed to surgical treatment after the 1-week follow-up in 1 patient and 1 patient was treated surgically after 3 months due to non-union (Fig. 4).

For all of the non-surgically treated patients, the treatment was converted to surgical treatment at an early stage in 5 patients (1%). In 2 of these patients, a slight lateralization of the talus at the 1-week follow-up was found and stability tests in the operating theatre confirmed what were regarded as unstable fractures. For 3 of the patients, however, the medical charts and radiographs did not reveal any clear indication for converting to surgical treatment. In addition, 2 of the non-surgically treated patients were treated surgically at a later stage due to non-union.

In the vast majority of cases (97%), plain radiographs were the only radiographic imaging modality used. During the treatment and follow-up period, the mean total number of radiographs performed for non-surgically treated patients were 2.2, compared with 2.9 in the surgically treated group.

27% of all B1 fractures (73% of the surgically treated and 8% of the non-surgically treated) were admitted to hospital. The surgically treated patients had an average of 4.0 outpatient visits, including the initial visit to the accident & emergency department (A&E) and the visit for outpatient surgery, and had an average of 2.6 days of inpatient care. 27% of the surgically treated patients had outpatient surgery with discharge the same day. The non-surgically treated patients had an average of 3.3 outpatient visits, including the initial visit to the A&E, and this group had an average of 0.2 days of inpatient care.

The surgical treatment performed for the B1 fracture was open reduction and internal fixation with a fibular plate in 65% and fibular plating combined with the fixation of the syndesmosis in 31%.

The average number of days of immobilization in a plaster or a brace were 46 for the surgically treated patients compared with 43 days for non-surgically treated patients. The difference in immobilization time corresponds well to the average 4.6 days that the surgically treated patients had to wait for their surgery.

In addition to the postoperative immobilization in a cast, 79% of the surgically treated patients and 50% of the non-surgically treated patients had some kind of weight-bearing restriction.

## Discussion

The current study describes the epidemiology of ankle fractures based on 1328 consecutive patients registered prospectively in the SFR during a 2-year period. The current study also makes a deeper analysis of the way lateral malleolar fractures, AO/OTA 44B1, were examined, classified and treated over the same time period at one of Sweden’s largest orthopedic centers.

Regarding the epidemiology of all types of ankle fracture, our main findings are in line with the findings of earlier studies [7]. We found that ankle fractures are common in all age groups and both sexes. The more severe, bimalleolar fractures tend to affect young men, whereas the unimalleolar fractures affect the older age group and predominantly women.

The classification of malleolar fractures, as well as treatment of them, is a matter of debate. At the time of the study there were no strict guidelines at the department regarding which fractures to treat surgically and which to treat non-surgically. The treatment plan was based on common knowledge in the field and the decision to treat surgically or not was made by the attending surgeon. We found that, even within the same department, there was some variation regarding the classification of fractures, the chosen treatment and subsequent weight-bearing restrictions, reflecting the lack of consensus in this area.

Among the 439 patients in the current study classified as having sustained an AO/OTA 44B1 fracture, 31% presented with medial tenderness at the physical examination. According to the AO/OTA classification, a trans-syndesmotic fracture of the lateral malleolus, combined with a rupture of the medial ligament, is a 44B2 fracture. Consequently, by definition, a 44B1 fracture has no significant rupture of the medial ligaments. A considerable number of these patients with medial tenderness, especially the ones where the responsible physician decided to treat surgically, can be suspected of having had a medial ligament injury and were not classified correctly. On the other hand, of all the B1 fractures treated non-surgically, despite having medial tenderness, in only 1 case the treatment was changed to surgical at an early stage. This could be interpreted as either indicating that the finding of medial tenderness was not equal to a significant structural medial ligament injury or that some B2 fractures can be treated non-surgically.

There is an apparent difficulty in clinically determining whether or not a lateral malleolar fracture has an associated injury to the medial ligament. Clinical signs like medial tenderness, ecchymosis and swelling are commonly considered, as well as tests for stability like the external rotation test [17] or stress gravity radiographs [18]. Since this fact determines whether the fracture is a stable B1 fracture or an unstable B2 fracture, it affects both the treatment of choice and the reliability of the fracture classification. In the current study the presence or absence of medial tenderness did not appear to be the deciding factor in the choice between surgical or non-surgical treatment, possibly due to the difficulty involved in evaluating clinically if a medial ligament injury is to be suspected or not. If a lateral malleolar fracture is classified as B1, despite having an injury to the medial structures, and undergoes surgical treatment, there is clearly a lack of understanding of the AO/OTA-fracture classification but a possible correct treatment. On the other hand, if a stable isolated lateral malleolar fracture with no medial injury is correctly classified as B1 and still undergoes surgical treatment, it is probably exposed to unnecessary surgical treatment and the risks that entails.

There is evidence supporting the benign course of B1 fractures. Pakarinen HJ et al. found that non-surgically treated patients had less pain and a better functional score compared with surgically treated patients [19]. They produced an algorithm based on stability to be used in decision-making to find the appropriate method of treatment for ankle fractures. In their study, 75% of the isolated lateral malleolar fractures (Weber type A or B) were treated non-surgically. None of the non-surgically treated patients had late displacement or needed surgical treatment during the 2-year follow-up period. In contrast to this, in the current study, 130 patients (30%) classified as having sustained a B1 fracture were treated surgically. This probably reflects a lack of understanding of the classification of ankle fractures. Despite this, we think this is an important finding, since the data in the current study were extracted from a national quality register used by clinicians and thereby represent real life.

Jain N et al. implemented guidelines aiming to diagnose stable ankle fractures reliably and manage them with no follow-up radiographs [20]. The guidelines for diagnosis and management were adopted from the work done by Wykes et al. [21]. Stable ankle fracture was defined as an undisplaced isolated lateral malleolar fracture with no medial tenderness or swelling. The fracture also had to be due to low/medium energy and to be a closed injury. The fracture had to be at or below the inferior tibiofibular joint and there should be no medial/posterior malleolar fracture. The patients were exclusively treated non-surgically, 91% in a brace, and they were all allowed immediate protected weight-bearing and full weight-bearing after 1 week. More than 80% were clinically healed after 4 weeks and no patients with secondary displacement were found.

In the current study, 79% of the surgically treated B1 patients and 50% of the non-surgically treated patients had some kind of weight-bearing restriction in addition to immobilization. If fixed surgically on the lateral side, a potentially unstable B fracture is transformed to a stable fracture and, in the authors’ opinion, should be allowed full weight-bearing postoperatively. The same goes for stable B fractures which, in the authors’ opinion, with the support of previously mentioned studies, should be treated non-surgically and allowed full weight-bearing.

Michelson et al. reviewed 82 non-surgically treated lateral malleolar fractures, followed by repeated radiographs, and found no secondary displacement at any stage [22]. As suggested by Michelson et al. and demonstrated by Jain N et al., significant financial savings can be attained if patients treated non-surgically do not have to undergo follow-up radiographic examination and are subject to fewer outpatient visits.

In the current study of non-surgically treated B1 fractures, the follow-up radiographic examination at 1 week changed the treatment plan correctly in only 3 cases (< 1%). This indicates that there is no need to check these fractures with radiographs at 1 week as a matter of routine, saving money, time and radiation. One limitation in the current study is, however, the lack of a long-term follow up, most importantly for the non-surgically treated “B1 fractures” with medial tenderness with respect to displacement and a subsequent need for surgery.

Another limitation of the current study is that it only includes patients treated at Sahlgrenska University Hospital. However, since this is Sweden’s largest orthopedic center, with a primary catchment area of approximately 600,000 inhabitants, we believe that it is representative of the population of Sweden.

The strengths of the current study are that it includes a large number of consecutive patients where data were collected prospectively. In comparison to earlier studies, it also includes patients that were hospitalized, as well as those treated as out-patients. Two years were studied and the results are not affected by seasonal variations. The results of the study reveal the application of the AO/OTA classification and real-life treatment algorithms.

The results of Michelson et al., Jain et al., Wykes et al. and the current study further support the hypothesis that, with the right guidelines strictly to distinguish stable from unstable ankle fractures, the stable fractures can be successfully treated non-surgically, with full weight-bearing allowed and with no need for follow-up radiographs. This would minimize the likelihood of patients being exposed to the risks of surgery with a fracture that could just as well be treated non-surgically. It would also minimize the number of radiographs per patient, reducing both costs and unnecessary risks. We believe that an algorithm that allows early weight-bearing would reduce patient suffering and allow for an earlier return to physical activity and work, beneficial both for the individual and for society. The results of the current study can be utilized to improve the treatment for this very common type of ankle fracture and to render the more effective use of healthcare resources.

## Conclusions

The current study demonstrates the difficulty involved in distinguishing whether or not a trans-syndesmotic lateral malleolar fracture has an associated medial ligament injury or not. As this distinguishes if the fracture is stable or not it affects the choice of subsequent treatment. The results of the study also indicate a lack of consensus on how to classify and treat lateral malleolar fractures at the level of the syndesmosis. The study further suggests that there is no need to check non-surgically treated stable fractures with follow-up radiographic examination at 1 week.

## Data Availability

The datasets used and analysed during the current study are available from the corresponding author on reasonable request.

## Abbreviations

SU: Sahlgrenska University Hospital
AO: Arbeitsgemeinschaft für Osteosynthesefragen
OTA: Orthopaedic Trauma Association
SFR: Swedish Fracture Register
SER: Supination-External Rotation
A&E: Accident & Emergency department
